# Are the Responses Generated Using a Rating Scale Or a Psychophysical Method Affected by Previously Presented Stimuli?

**DOI:** 10.1068/ig13

**Published:** 2013-10-01

**Authors:** S C M Quinn, E Lazare, P J B Hancock, R J Watt

**Affiliations:** University of Dundee, UK; University of Stirling, UK; University of Stirling, UK; University of Stirling, UK

## Abstract

In most studies, participants are presented with numerous trials to complete. Whilst this method is often used in experimental research, it has been criticised because previous exposure to the stimulus set could bias future responses (Warren, 1970). The only response that is unaffected by previously presented stimuli is the response given on the first trial. In making a single response to one stimulus, any possibility of biasing future responses would be eliminated. If the pooled responses of many participants, each responding to a single stimulus, generated similar results to an experiment where a group of participants responded to the full range of stimuli, it would demonstrate that responses are not affected by previously presented stimuli. In a recent auditory psychophysical study, we showed that the responses generated by listeners, each responding to a single stimulus presentation, are essentially similar to those involving multiple responses (Quinn and Watt, 2012). This suggests that listeners' responses are essentially independent of previously presented stimuli. In four experiments, we compare single stimulus responses with multi-stimulus responses where observers are asked to judge the attractiveness of faces on a rating scale (experiments 1 and 3) or using a 2AFC method (experiments 2 and 4). Results are discussed in terms of the information content of each method. [Supported by The Nuffield Foundation]

